# Intestinal Epithelium Modulates Macrophage Response to Gliadin in Celiac Disease

**DOI:** 10.3389/fnut.2019.00167

**Published:** 2019-11-05

**Authors:** Gloria Serena, Daniel Huynh, Rosiane S. Lima, Luciana M. Vise, Rachel Freire, Laura Ingano, Maureen M. Leonard, Stefania Senger, Alessio Fasano

**Affiliations:** ^1^Division of Pediatric Gastroenterology and Nutrition, Center for Celiac Research, Mucosal Immunology and Biology Research Center, Massachusetts General Hospital, Boston, MA, United States; ^2^Harvard Medical School, Boston, MA, United States; ^3^European Biomedical Research Institute of Salerno, Salerno, Italy

**Keywords:** celiac disease, macrophages, epithelium, gliadin, innate immunity

## Abstract

Celiac disease is an immune-mediated enteropathy triggered by ingestion of gluten. Although its pathogenesis has been extensively studied and the contribution from both innate and adaptive immune responses has been reported, little is still known about the contribution of macrophages to the onset or maintenance of the disease. Macrophages are extremely plastic immune cells that can be directed toward a pro- or anti-inflammatory phenotype by the surrounding microenvironment. Of note, gliadin, the most prominent causative agent of the disease, has been reported to trigger the production of pro-inflammatory cytokines in this cell population. In the present study, we aimed at investigating how the intestinal milieu and more specifically the epithelium can shape the macrophage response to gliadin. Using patient-derived organoids we showed that the intestinal epithelium derived from celiac disease donors releases anti-inflammatory factors that curb the macrophage response to gliadin. Furthermore, we uncovered that the celiac macrophages were better responders than macrophages derived from non-celiac controls. Finally, we demonstrated that IFNγ released by the epithelium is in part responsible of the observed anti-inflammatory effect. Our data shed light on the cross–talk between the immune system and the epithelium and its critical role in the intestinal homeostasis. Furthermore, we provide more evidence how alterations in the innate immune machinery in celiac patients may contribute to the onset of the disease.

## Introduction

Celiac disease (CD) is an immune-mediated enteropathy that affects about 1% of the general population worldwide (1). It is triggered by ingestion of gluten, a composite protein made of gliadin and glutenin proteins (1). An altered intestinal epithelial barrier (2), specific genetic predisposition (3), chronic inflammatory immune response to gliadin (4) and its deficient suppression by Treg cells (5) are the main known elements that contribute to its onset.

The inflammatory process underling CD pathogenesis involves the activation of the innate as well as the adaptive immune system (6). Relatively to the adaptive component, CD pathogenesis is predominantly driven by a strong Th1 immune response with production of pro-inflammatory cytokines, such as interferon gamma (IFNγ) and tumor necrosis factor alpha (TNFα) (7). The contribution of Th17 cells to CD development has also been recently established, as interleukin 17 A (IL17A) was found significantly more expressed in CD patients (8, 9).

While the adaptive immune response plays a fundamental role in establishing a chronic inflammation, the innate immune machinery drives the initial steps of the inflammatory cascade (10). Production of innate pro-inflammatory cytokines, such as interleukin 15 (IL15) (11–13) and interferon alpha (IFNα) (14) has been reported in untreated CD patients upon ingestion of gliadin. Interleukin 8 (IL8), a potent neutrophils chemoattractant, has been shown to be released by the epithelium and immune cells of CD patients (15) and specific gluten peptides have been reported to have chemo-attractant properties as well (16, 17).

Macrophages (MΦ) are key modulators and effectors of the immune response (18, 19). They protect against pathogens or toxic endogenous elements and act as antigen presenting cells to other immune cells (18, 19). Their inherent plasticity allows them to respond to a variety of environmental stimuli by acquiring either a pro-inflammatory (M1) or an anti-inflammatory (M2) phenotype (18, 20). In the intestinal lamina propria, the majority of MΦ originates from circulating monocytes recruited to the tissue and differentiated into MΦ under the influence of the surrounding microenvironment (19).

The contribution of MΦ to the pathogenesis of CD has been previously suggested (21–25). *In vitro* studies with murine MΦ and human monocytic cell lines have shown that gliadin triggers production of TNFα, IL8, RANTES, interleukin 1β (IL1β) and significantly increases nitric oxide (NO) upon activation of toll-like receptors 2 and 4 (TLR2/TLR4) (9, 11, 21–23). To our knowledge, however, studies investigating the effect of gliadin on human primary MΦ are scarce.

In this study, we evaluated the response of primary human monocytes derived MΦ to gliadin. Furthermore, using human intestinal derived organoids, we studied the contribution of the epithelium in modulating MΦ phenotype and function.

Our data show that gliadin triggers a potent inflammatory response from human primary MΦ and that the intestinal epithelium from patients with CD down-regulates MΦ's response to gliadin. Our experiments also demonstrate that MΦ from patients with CD are more responsive to epithelium-derived signals than MΦ from non-CD subjects. These findings highlight the importance of the cross talk between the intestinal epithelium and immune cells during the early phase of CD and underline alterations in the innate immune machinery of CD patients that may fundamentally contribute to the loss of tolerance to gluten.

## Materials and Methods

### Human Subjects

Whole blood was obtained by venipuncture from adult patients aged between 14 and 65 years old during routine visits to our clinic at Massachusetts General Hospital. For the experiments with monocytes two groups of patients were recruited: non-celiac healthy control subjects (HC) and patients with CD in remission following a gluten free diet (CDGF). Patients were considered in remission state if, at the time of blood withdraw, they have been following a gluten free diet for at least 6 months and presented negative serology and normal intestinal mucosa (Marsh 0 to II). For the experiments with fresh biopsies three groups of patients were recruited: healthy controls (HC), celiac patients in remission (CDGF) and active celiac patients (CDA). Biopsies were collected during clinically-indicated upper endoscopic procedures. Patients with CDA in the cohort were diagnosed based on pathological evaluation (Marsh III at time of diagnosis) and positive serology results for anti-human tissue transglutaminase IgA antibodies (INOVA Diagnostic) (manufacturer instructions were followed and samples were evaluated positive if values of TtG-IgA ≥20 were detected). Finally, for the experiments with supernatants derived from primary epithelia, we used organoids from our biorepository that were previously generated from small intestinal biopsies of active CD patients (CDA *n* = 4), CD patients in remission (CDGF *n* = 1), and healthy controls (HC *n* = 5). Because our previous study has shown no functional difference between the celiac derived organoids (active and in remission) (15), they were grouped together and labeled as CD.

### Monocytes Isolation and Differentiation

Peripheral blood mononuclear cells (PBMC) were isolated from whole blood samples by density gradient centrifugation over Ficoll (GE Healthcare). Monocytes CD14^+^ were isolated from the PBMC population using CD14^+^ magnetic beads (MACS) and cultured for 7 days in complete RPMI medium [RPMI 1640 Dutch modification (Gibco), supplemented with 10% FBS (Sigma-Aldrich), 1% L-glutamine, 1% penicilin/streptomycin, 1% sodium pyruvate, and 1% non essential amino acid solution (Gibco)] and 50 ng/ml of M-CSF (Peprotech) to induce MΦ differentiation. Medium was changed every 3 days. At day 7 monocytes derived MΦ were stimulated.

### Organoids Establishment From Duodenal Crypts

Human intestinal organoids from small intestinal biopsies were previously established from both CD patients and non celiac healthy donors (HC) and monolayers were generated as previously described (15). Briefly duodenal biopsies were stored in ice cold medium and epithelial cells were isolated with EDTA dissociation buffer. Intenstinal crypt were cultured in matrigel as previously described (15) to generate intestinal organoids. The cultures were then passaged every 7 days using trypsin-based method of dissociation and the appropriate number of single cells were re-plated in matrigel with LWR-N/ISC medium until passage 22 as previously described (15). A total of *n* = 5 CD and *n* = 5 HC control derived monolayers were employed in this study at comparable passage.

### Macrophages Stimulation

At day 7 MΦ were stimulated for 24 h with different conditions depending on the experiment as follow. Pepsin-trypsin digested gliadin (PTG) was prepared as previously described (9, 26) and tested for endotoxin level. MΦ were stimulated for 24 h with 1 mg/ml of PTG in complete RPMI medium.

To study epithelial contribution to MΦ's response to gliadin, derived human intestinal organoids monolayers from HC and CD patients were apically stimulated for 4 h with 1 mg/ml of PTG as previously described (15). Organoids monolayers' basolateral supernatants were then used to stimulate MΦ for 24 h or 30 min depending on the experiment.

To investigate the role of IFNγ in MΦ response to PTG, we stimulated MΦ from CD patients for 24 h with basolateral supernatants from both HC and CD organoids treated with PTG to which we respectively added recombinant IFNγ (20 ng/ml) (Thermo Fisher Scientific), anti-IFNγ blocking antibody (10 μg/ml) (Thermo Fisher Scientific), or Mouse IgG1k isotype (10 μg/ml) (Thermo Fisher Scientific).

### Isolation of Immune Cells From Small Intestinal Biopsies

For each patient between two and four biopsies were collected and placed in ice-cold RPMI 1640 complete medium enriched with 10% FBS. The specimens were then incubated for 20 min at room temperature in calcium and magnesium free HBSS 1X (Gibco) and 1 mM DTT to eliminate mucus in excess. Biopsies were incubated at 37°C for 30 min under tilting in calcium and magnesium free HBSS 1X and 1 mM EDTA (Ambion) to remove the epithelium and followed by two brief washes in calcium and magnesium free HBSS 1X. The intestinal lamina propria was then incubated for 30 min at 37°C under tilting with RPMI 1640 complete medium enriched with 2% FBS containing 20 μg/ml of DNAse I (Roche) and 1 mg/ml of collagenase (Sigma). After digestion mononuclear immune cells were isolated by Ficoll.

### Gene Expression Analysis

Total RNA was extracted from intestinal biopsies and monocytes derived MΦ, respectively by using trizol as previously described (5) and RNeasy Extraction micro kit (Qiagen). Reverse transcription was performed using RevertAid First Strand cDNA Synthesis Kit (Fermentas) followed by Real-Time PCR using PerfeCta SYBR Green SuperMix (Quanta Bioscences). Specific human primers for 18S, CD68, TNFα, IL6, IL1β, and TGFβ were used to evaluate gene expression (Table 1). Cycling conditions were 10 min at 95°C, followed by 40 cycles of 15 s at 95°C, 30 s at 60°C and 30 s at 72°C, finally 1 min at 95°C and 1 min at 65°C.

**Table 1:** Sequences for forward and reverse primers used to measure gene expression by real-time RT-PCR.

**Gene**	**Sequence primers**
18S	*FORWARD* 5′-AGAAACGGCTACCACATCCA-3′ *REVERSE* 3′-CCCTCCAATGGATCCTCGTT-5′
CD68	*FORWARD* 5′-CAGCCTAGCTGGACTTTGGG-3′ *REVERSE* 3′-GGGTGTCACCGTGAAGGATG-5′
TNFα	*FORWARD* 5′-CAAGGACAGCAGAGGACCAG-3′ *REVERSE* 3′-TGGCGTCTGAGGGTTGTTTT-5′
IL6	*FORWARD* 5′-CCAGAGCTGTGCAGATGAGTA-3′ *REVERSE* 3′-TTGGGTCAGGGGTGGTTATTG-5′
IL1β	*FORWARD* 5′-GGCTGCTCTGGGATTCTCTT-3′ *REVERSE* 3′-CCATCATTTCACTGGCGAGC-5′
TGFβ	*FORWARD* 5′-TGCGCTTGAGATCTTCAAA-3′ *REVERSE* 3′-GGGCTAGTCGCACAGACCT-5′

### ELISA

ELISA assay was performed on supernatants from MΦ at baseline and after PTG stimulation. TNFα, IL6, and IL1β production was assessed by using BD Opteia human kit (BD bioscience) following manufacturer's instructions.

### Flow-Cytometry

The presence of MΦ in the intestinal lamina propria of CDA or HC subjects and the phenotype of monocytes derived MΦ was evaluated by flow cytometry as follow: MΦ were stained with antibodies anti-CD80 (BD bioscience) and CD206 (BD bioscience) to evaluate the percentages of classically activated pro-inflammatory (M1) and alternatively activated anti-inflammatory (M2) MΦ, respectively; CD 68 antibody was used as a pan marker for MΦ. Activation of IFNγR and pSTAT1 on monocytes derived MΦ was evaluated using IFNγR (Thermofisher) and intracellular pSTAT1 (Ser767) (Abcam) antibodies after methanol permeabilization. Data were acquired with BD FACS Calibur flow cytometer instrument and analyzed with BD CellQuestPro and FlowJo softwares.

### Statistics

Statistical analysis of the samples was performed by *t*-test, two-tailed Mann-Whitney test (unpaired samples) and paired *t*-test or Wilcoxon test (paired samples) as appropriate. Standard error of the mean (SEM) was measured to determine the dispersion among data within the same group. In all statistical tests, a *p*-value < 0.05 was considered significant.

### Study Approval

All protocols were approved by the Massachusetts General Hospital Partners Human Research Committee Institutional Review Board and written informed consent was obtained from all enrolled subjects (2014P000198, 2013P000288).

## Results

### Macrophages Are Increased in Active Celiac Disease

**Figure 1:**
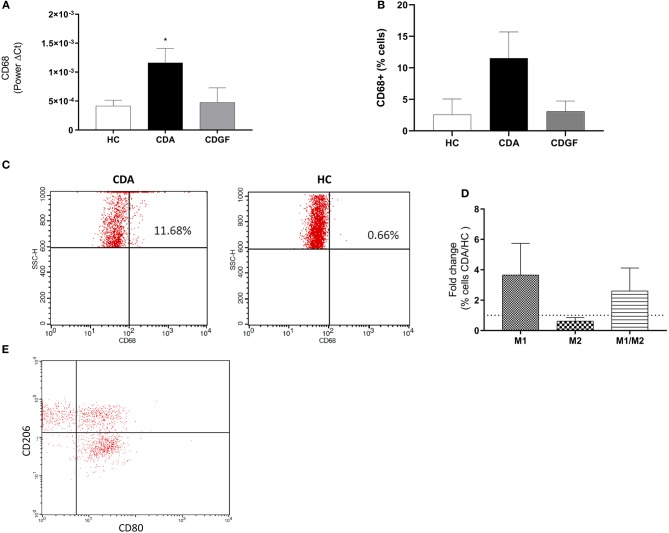
Macrophages phenotype in small intestinal biopsies of celiac patients. **(A)** Gene expression analysis of CD68, a pan marker for macrophages (MΦ), in small intestinal biopsies from healthy controls (HC *n* = 12), active celiac patients (CDA *n* = 17), and celiac patients in remission (CDGF *n* = 7). Real-time RT-PCR data were normalized to housekeeping gene 18S. **(B)** Percentage of total CD68^+^ MΦ in small intestinal lamina propria of HC (*n* = 4), CDA (*n* = 4) patients and celiac patients in remission (CDGF *n* = 3). **(C)** Representative gating strategy to calculate percentage of CD68^+^ cells in small intestinal biopsies of HC and CDA patients. **(D)** Percentage of M1 (CD80^+^), M2 (CD206^+^), and M1/M2 (CD80^+^CD206^+^) phenotypes in small intestinal lamina propria of HC (*n* = 4) and CDA (*n* = 4) patients represented as fold change over non-celiac healthy control group. **(E)** Representative gating strategy to calculate percentage of CD68^+^, M1, M2, M1/M2 cells in small intestinal biopsies of HC and CDA patients. CD80^+^ and CD206^+^ cells were calculated within the CD68^+^ cells gate. Statistical analysis was calculated using Mann–Whitney *t*-test. *P < 0.05.

To investigate the contribution of MΦ to CD pathogenesis, we measured the gene expression of CD68, an intracellular MΦ pan-marker in duodenal biopsies from HC, CDGF, and CDA patients. We observed an increased expression of CD68 in CDA as compared to HC (Figure 1A), suggesting an augmented frequency of this cell type in active untreated CD patients. Whereas, the expression of CD68 in CDGF biopsies was comparable to HC (Figure 1A). Flow-cytometry analysis of mononuclear immune cells isolated from the lamina propria of intestinal biopsies, further confirmed the higher abundance of total MΦ in CDA patients when compared to HC (Figures 1B,C) and comparable percentage of CD68^+^ cells between HC and CDGF (Figure 1B). Immune phenotyping of the macrophage population revealed a slight increase, though not significant, of both M1 and M1/M2 populations in CDA macrophages (Figures 1B–E).

Overall the increased percentage of MΦ in the lamina propria of active CD patients suggests that these cells may play a role in the inflammatory process driving CD pathogenesis.

### Gliadin Triggers a Pro-inflammatory Phenotype in Human Primary Macrophages

**Figure 2:**
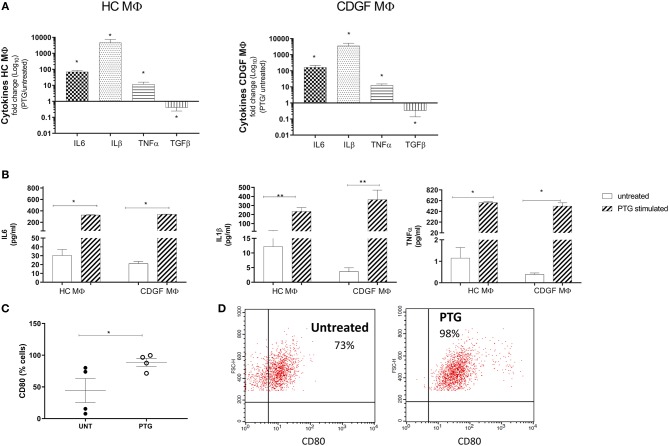
Gliadin triggers a pro-inflammatory response in human primary macrophages. **(A)** Gene expression for pro- and anti-inflammatory cytokines in human primary macrophages (MΦ) from healthy (HC *n* = 8) and celiac (CDGF *n* = 7) patients stimulated with 1 mg/ml of pepsin-trypsin digested gliadin (PTG). Each bar represents the fold change over untreated control. Real-time RT-qPCR data were normalized to housekeeping gene 18S. **(B)** Quantification of cytokines secreted by CDGF and HC MΦ untreated (white bars) or stimulated with PTG (stripe bars). **(C)** Percentage of pro-inflammatory M1 cells (CD80^+^) out of total MΦ from CDGF patients (*n* = 4) untreated (UNT) or stimulated with 1 mg/ml of PTG (PTG). **(D)** Gating strategy to detect CD80^+^ cells in MΦ untreated or stimulated with PTG. CD80^+^ cells were gated out of cells that were considered activated MΦ depending on FSC and SSC values. The experiment is representative of the experiment (*n* = 4). Statistical analysis was calculated using Mann–Whitney *t*-test. *P < 0.05; **P < 0.005.

To assess the effect of gliadin on human primary MΦ, we stimulated monocytes-derived MΦ from HC and CDGF patients with pepsin-trypsin digested gliadin (PTG) that had been previously tested negative for endotoxin levels (data not shown). Macrophages were derived from CD patients in remission because they have not been previously exposed to gliadin and therefore better represented cells that first encounter the external antigen. PTG triggered a pro-inflammatory response in MΦ from both experimental groups, upregulating the expression of M1-specific cytokines, such as IL6, IL1β, TNFα, and downregulating the expression of M2-specific TGFβ (Figures 2A,B; Supplementary Figure 1). Flow-cytometry analysis confirmed the functional shift of MΦ upon stimulation with PTG toward a pro-inflammatory phenotype characterized by increased number of M1 CD80^+^ cells (Figures 2C,D).

Taken together, these findings further support previous observations on the pro-inflammatory effect of gliadin on MΦ and other cell types (15, 17) independently on the disease status.

### Celiac Epithelium Modulates Macrophages Response to Gliadin

**Figure 3:**
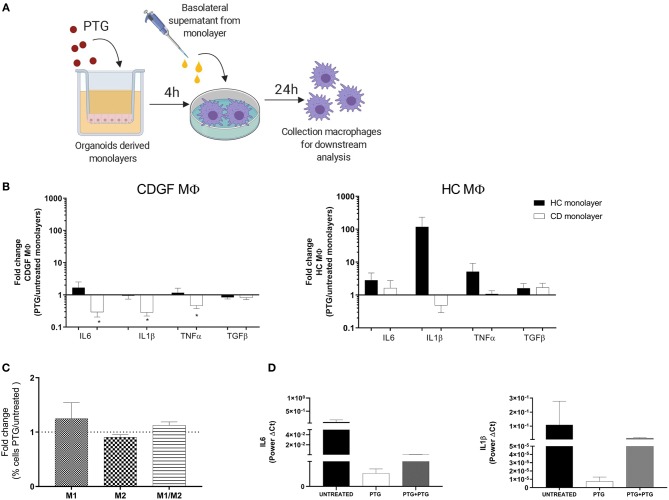
Celiac epithelium influences macrophages response to gliadin. **(A)** Experiment design to investigate effect of epithelium on MΦ response to gliadin: organoids derived monolayers from healthy controls (HC *n* = 5) and celiac patients (CD *n* = 5) were plated on transwells to form polarized monolayers and treated for 4 h with 1 mg/ml of pepsin-tripsyn gliadin (PTG). Basolateral supernatants were collected and used to stimulate for 24 h human primary MΦ differentiated from circulating monocytes from HC and CDGF patients (see Materials and Methods). Total RNA from MΦ was extracted for gene expression analysis. **(B)** Gene expression analysis of cytokines in monocytes derived MΦ isolated from HC (*n* = 7) and CDGF (*n* = 6) patients and *in vitro* stimulated, respectively with supernatants of monolayers from HC (black bars *n* = 5) and CD (white bars *n* = 5) organoids. Bars represent the fold change of MΦ treated with PTG stimulated organoids over the untreated ones. **(C)** Percentage of M1 (CD80^+^), M2 (CD206^+^), or M1/M2 (CD80^+^CD206^+^) phenotypes in MΦ from CDGF (*n* = 5) patients cultured with CD epithelium. Bars represent the fold change of MΦ treated with PTG stimulated epithelium over the untreated condition. **(D)** Gene expression of pro-inflammatory cytokines in MΦ from CDGF patients (*n* = 4) cultured with supernatants from untreated CD monolayers (black bars), monolayers stimulated with PTG (white bars), and monolayers stimulated with PTG and extra PTG added to the MΦ themselves (gray bars). All real-time RT-PCR data were normalized to housekeeping gene 18S. Statistical analysis was calculated using paired Wilcoxon test. *P < 0.05.

MΦ are very plastic cells whose phenotype and function are continuously shaped by the surrounding microenvironment (18). Epithelial cells are a major source of stimuli, playing an important role in the immune response modulation (27). Furthermore, the epithelial cells act as a physical barrier between the inner and outer world and consequently are the first cells to get in contact with and react to any foreign agents. In order to investigate the response of MΦ to gliadin in a more physiologically relevant milieu we sought to evaluate how the epithelium influences the response of MΦ to gliadin. We stimulated MΦ from HC and CD patients with the basolateral supernatants from HC and CD cell monolayers that had been previously apically exposed to PTG and assessed cytokines gene expression in the MΦ population (Figure 3A). Supernatants from CD epithelium had an overall anti-inflammatory effect on CD MΦ characterized by a significant decrease in TNFα, IL6, and IL1β expression when compared to CD macrophages treated with the same monolayer-derived supernatants at baseline. Conversely, HC epithelium did not alter the MΦ response to gliadin (Figure 3B). HC MΦ mirrored the response of CD MΦ to the different epithelia-derived milieu, however, they were less responsive (Figure 3B). Importantly, regardless of the change in pro-inflammatory cytokines production, the overall phenotype of MΦ did not significantly change showing only a minor, but non-significant increase of M1 and double positive M1/M2 cells (Figure 3C). Our data, in fact, show that the percentage of M1 and M1/M2 cells expressed as ratio between MΦ stimulated with supernatants from PTG treated organoids and untreated organoids is only slightly above 1.

We have previously reported that gliadin triggers disruption of intestinal epithelial barrier function in CD, but not in HC (15, 28). Based on our experimental design, we expected that MΦ will be exposed to PTG that might have crossed the cell monolayer, as well as to factors released by the epithelium itself. To further investigate the anti-inflammatory properties of CD epithelium on MΦ after PTG stimulation, we cultured CD MΦ with conditional media from CD organoids pre-treated with PTG and we added 1 mg/ml of PTG directly to the MΦ. Although not significant, our data showed that even after addition of extra PTG on MΦ, supernatants from PTG stimulated CD epithelium were still reducing IL6 and IL1β expression (Figure 3D). Interestingly TNFα expression did not follow the same decrease, therefore suggesting an independent mechanism for its regulation (data not shown).

These data highlight differences between CD and HC MΦ and show for the first time that MΦ response to gliadin is specifically influenced by the intestinal epithelium.

### Epithelium Derived IFNγ Modulates Macrophages Response to Gliadin

CD epithelium releases multiple pro-inflammatory cytokines upon exposure to gliadin (28–30). Our group has recently shown that cytokines (15), including IL6, IL15, and TNFα, and IFNγ are released by organoids after stimulation with gliadin. Our data also confirmed that, upon stimulation with PTG, CD organoids produced increased amount of IFNγ (Supplementary Figure 2A). This is one of the most predominant cytokines secreted by the immune cells in CD (31) and play a major role in the Th1 driven immune response (32, 33). It has been shown to have pleiotropic properties and to play prominent role in regulating MΦ's function (34, 35). Based on these findings we aimed at assessing whether its release by CD epithelium may be responsible for modulating MΦ response to gliadin.

**Figure 4:**
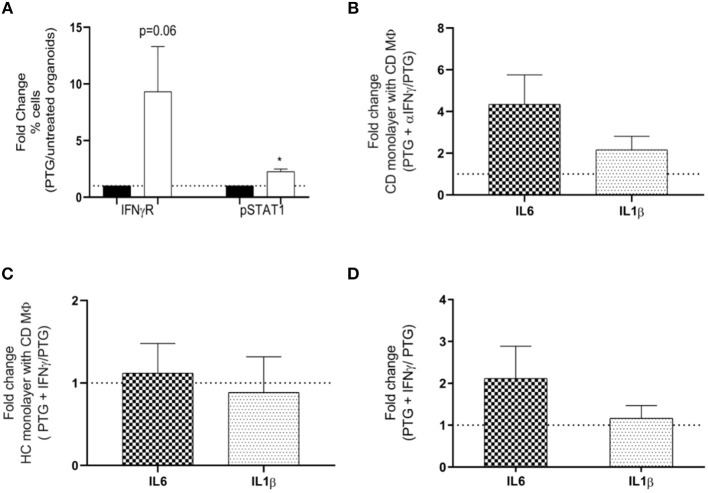
IFNγ released by the epithelium modulates macrophages response to gliadin in CD. **(A)** Flow-cytometry analysis evaluating activation of IFNγ receptor (IFNγR) pathway by calculating expression of IFNγR and phosphorylation of its downstream partner STAT1 (pSTAT1). Bars represent fold change of MΦ from CDGF patients (*n* = 5) treated with supernatants derived from CD organoids stimulated with PTG (white bars) compared to untreated (black bars). **(B)** Gene expression analysis of IL6 and IL1β in monocyte derived MΦ from CDGF patients (*n* = 8). Each bars represents fold change of MΦ stimulated with supernatants from CD monolayers treated with PTG and αIFNγ over the cells stimulated only with the PTG treated CD supernatant. **(C)** Gene expression analysis for IL6 and IL1β in MΦ from CDGF patients (*n* = 9). Each bar represents fold change of MΦ stimulated with supernatants from HC organoids treated with PTG and IFNγ over the cells stimulated only with the PTG treated HC supernatant. **(D)** Gene expression analysis of IL6 and IL1β in MΦ from CDGF patients (*n* = 3). Each bar represents fold change of MΦ stimulated PTG and IFNγ over the cells stimulated only with PTG. All real-time RT-PCR data were normalized to housekeeping gene 18S. Statistical analysis was calculated using paired Wilcoxon. *P < 0.05.

Using flow-cytometry analysis we measured the activation of the IFNγ receptor (IFNγR) pathway in MΦ stimulated with supernatants from CD. Our data showed that, after stimulation with PTG, supernatants from CD epithelium triggered an increased expression of IFNγR and activation of its signaling partner pSTAT1 in MΦ (Figure 4A). These findings suggest that IFNγ released by the epithelium and therefore present in the supernatants of CD organoids may be one of the factors responsible for the increased activation of IFNγ receptor. Further studies are needed to exclude the contribution of other components. We further confirmed the contribution of IFNγ in down-regulating the expression of selected cytokines in MΦ, by culturing CD MΦ with supernatants from CD organoids treated with PTG and α-IFNγ antibody. Blocking IFNγ in CD supernatants showed a reduced trend of their anti-inflammatory properties causing a 4 times fold increase of IL6 and a 2 times fold increase of IL1β (Figure 4B).

The addition of recombinant IFNγ to supernatants from HC organoids, however, was not sufficient to trigger a reduction of IL6 or IL1β in CD MΦ (Figure 4C and Supplementary Figure 2B). Furthermore, IFNγ did not decrease the inflammatory response in MΦ stimulated directly with PTG (Figure 4D).

These data combined suggest that IFNγ released by CD epithelium following PTG stimulation is necessary but not sufficient to down-regulate the pro-inflammatory response of CD MΦ to PTG.

## Discussion

The role of innate immune response in CD pathogenesis has been suggested for long time. Innate immune cytokines, such as IL15 and IFNα have been shown to contribute to the loss of tolerance to gluten (14) and various innate immune cells have been proposed to contribute to earlier steps leading to loss of tolerance to gluten (14). MΦ are unique cells that play a major role in the innate immune response machinery and create a bridge with the adaptive immunity by acting as antigen presenting cells (APC) (18).

The role of MΦ in CD pathogenesis has been suggested by numerous studies performed on monocytic human cell lines and murine macrophages showing that gliadin triggers an inflammatory response in these cells (9, 21, 22). However, research on the effect that gliadin exerts on human primary MΦ is limited.

As occurs for most of the immune cells, MΦ are very susceptible to the surrounding microenvironment that can drive them to acquire a pro-inflammatory M1 or anti-inflammatory M2 phenotype (20). Recently, another set of MΦ expressing both M1 and M2 markers have been described both in the healthy lung mucosa and in the circulation of systemic sclerosis patients (36). While the role of these double positive cells is still under debate, it has been suggested that the presence of both M1 and M2 features could confer to resident MΦ the ability to maintain a balance between immune tolerance and protective immunity (36).

Our data show that the environmental trigger for CD, gliadin, has a pro-inflammatory effect on human primary MΦ driving their differentiation toward an M1 phenotype. This effect was seen in both celiac and healthy MΦ. While these data confirm previously published data (21, 37), they are in contrast with recent findings showing MΦ taking an M2-like shift triggered by gliadin in CD patients (38). The conflicting findings may be explained by the different methodology used by the authors to generate differentiated MΦ. Furthermore, studying the effect of gliadin on isolated MΦ may not completely recapitulate the physiological encounter occurring in the gut. For this reason and given the important role that the surrounding micromilieu plays in modulating the immune system, we investigated the gut epithelium influence on MΦ response to gliadin. Our experiments using previously characterized organoids derived from patients diagnosed with CD revealed a strong anti-inflammatory effect of CD epithelium on MΦ. On the contrary, HC epithelium derived milieu, either increased or did not alter the pro-inflammatory cytokines expression of the MΦ, suggesting that the anti-inflammatory properties of CD epithelium could represent a genetic or acquired mechanism to compensate the inflammation triggered by gliadin.

Our data also show that CD MΦ are more responsive to the intestinal epithelium-derived microenvironment as compared to MΦ from HC patients. These findings are in line with our previous observations in CD derived Treg cells that responded differently to the same environment when compared to HC ones (5). Further studies investigating the effect of the epithelium on macrophages' response to gliadin in HC are needed to better understand the intrinsic differences between the two groups of samples. Additionally, studying how MΦ derived from CDA patients respond to gliadin, would provide some insights on the role that the epithelium plays during continuous exposure to gluten (acute phase).

Interestingly the overall MΦ phenotype in CD patients did not significantly change after gluten exposure, showing only a minor increase of M1 and double positive M1/M2 cells. The same pattern was seen in the biopsies from CDA patients, therefore suggesting that our model effectively reflects the *in vivo* state.

MΦ are extremely plastic cells and switch between phenotypes during which cells maintain both phenotypes have been previously reported (39). Given the presence of double positive M1/M2 phenotypes in the small intestinal biopsies of active CD patients, however, it is plausible to hypothesize that these cells represent a defined MΦ population rather than a transient one. The exact function of M1/M2 macrophages is not well defined yet. More studies are needed to accurately understand the contribution that these macrophages give to the overall CD development. We hypothesize that in response to the gliadin pro-inflammatory micromilieu, the macrophages differentiate toward a less inflammatory phenotype M1/M2 in order to return the mucosa to homeostatic condition.

Gliadin triggers numerous changes in the epithelium of CD patients (15) and a vast array of cytokines is produced by enterocytes, IFNγ being one of those. Consistent with previous observations, we confirmed that IFNγ is secreted by CD organoids after stimulation with PTG. IFNγ is a pleiotropic cytokine that can induce or down-regulate inflammation (35). Low doses of IFNγ have been shown to have anti-inflammatory effects in asthma and EAE animal models as well as in human (35, 40). Furthermore, IFNγ has been shown to be produced by a specific subset of regulatory T cells IL10/IFNγ Tr1 and to induce activation of *FOXP3* promoter gene in Treg cells (35, 41). Based on these findings, we investigated the contribution of IFNγ to the anti-inflammatory effect of gliadin-treated CD epithelium on MΦ. Our data showed that supernatants from CD gut epithelium activated the IFNγ receptor pathway in CD MΦ and that this cytokine was necessary but not sufficient to down-regulate MΦ response to gliadin. Our group has previously reported that some anti-inflammatory cytokines are upregulated in CD organoids and in CDA and CDGF biopsies (15). We can speculate that the epithelium might upregulate anti-inflammatory cytokines as a compensatory mechanism to inflammation driven by gliadin.

While our data have shown the fundamental contribution of IFNγ, open questions remain to be elucidated regarding the mechanisms through which this cytokine can modulate MΦ response to gliadin and whether other epithelium- derived components can contribute to this modulation.

**Figure 5:**
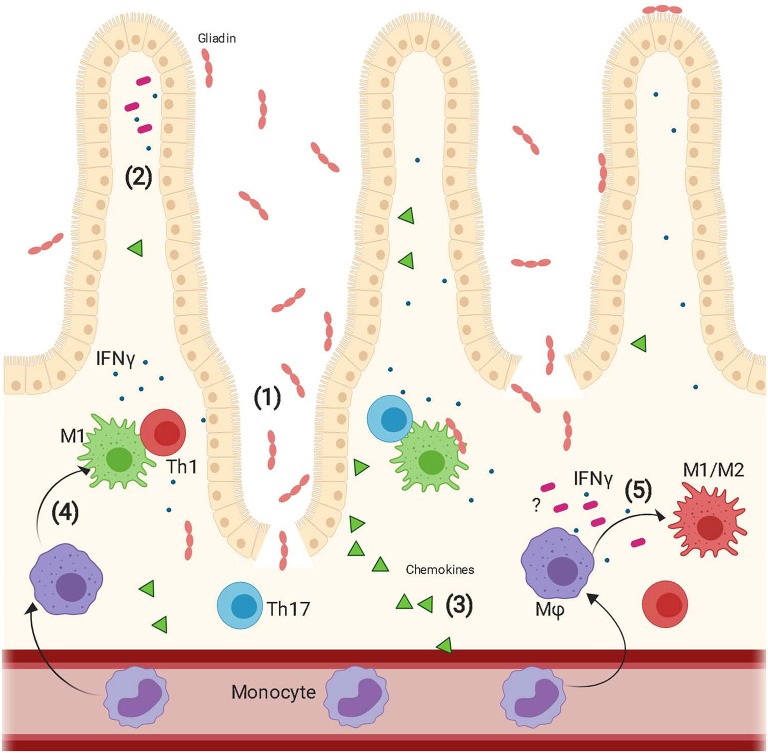
Proposed model of epithelium contribution to macrophages' response to gliadin. Upon ingestion, gliadin is partially digested in different peptides that interact with the epithelium triggering increased intestinal permeability (1) and secretion of a vast array of cytokines (2) and chemokines (3). Circulating monocytes are recruited to the intestinal lamina propria by the epithelium released chemokines and here they differentiate into macrophages. Gliadin peptides trigger activation of macrophages toward a pro-inflammatory M1 phenotype (4). These cells secrete pro-inflammatory cytokines and initiate a Th1/Th17 immune response. Additionally, recognition of gliadin peptides combined with the effect of IFNγ released from the epithelium and additional unknown epithelium derived cytokines triggers also differentiation of macrophages toward an M1/M2 phenotype whose function is still under study (5).

Overall this study highlights the importance of the interaction between innate immune cells and intestinal microenvironment in CD pathogenesis and suggests that an orchestrated response of multiple players including epithelium and MΦ to gliadin may fundamentally contribute to the development and progress of the disease (Figure 5).

## Data Availability Statement

The datasets generated for this study will not be made publicly available. The project is still ongoing.

## Ethics Statement

The studies involving human participants were reviewed and approved by Massachusetts General Hospital Partners Human Research Committee Institutional Review Board. Written informed consent to participate in this study was provided by the participants' legal guardian/next of kin.

## Author Contributions

GS developed the hypothesis, designed and performed the experiments, analyzed the data, and wrote the manuscript. DH, RF, and LI helped in performing the part of the experiments. RL, LV, and ML provided the clinical samples. AF and SS developed the overall hypothesis and supervised the research. All authors critically reviewed the manuscript.

### Conflict of Interest

The authors declare that the research was conducted in the absence of any commercial or financial relationships that could be construed as a potential conflict of interest.

## Supplementary Material

Supplementary Figure 1 **(A)** Representative example of CD14^+^ cells isolation purity. CD14^+^ cells (green pick) were isolated from PBMC by positive selection from the CD14^−^ cells (purple pick). **(B)** IL6 gene expression on MΦ from CDGF (*n* = 4) untreated (UNT), stimulated with pepsin-trypsin gliadin (PTG), or with negative control pepsin-tryspin digested water (PTH2O) represented as fold change over the untreated condition. IL6 was chosen as a representative cytokine to confirm the specific pro-inflammatory effect of gliadin. **(C)** Gene expression analysis of pro-inflammatory cytokines in MΦ from CDGF patients (*n* = 6) stimulated with increasing concentrations of PTG. Real-time RT-PCR data were normalized to housekeeping gene 18S. Statistical analysis was calculated using Friedman paired test. ^*^*P* < 0.05.

Supplementary Figure 2 **(A)** Production of IFNγ measured by ELISA in supernatants from HC (circles) and CD (triangles) organoids before and after stimulation with PTG. **(B)** Gene expression analysis for IL6 and IL1β in MΦ from CDGF patients (*n* = 3) stimulated with supernatants from CD organoids stimulated with PTG (black bars) and with αIFNγ (white bars) or correspondent isotype added (gray bars). Real-time RT–PCR data were normalized to housekeeping gene 18S.
